# Screening for Barrett’s esophagus and esophageal adenocarcinoma: recent advances and next steps

**DOI:** 10.1093/dote/doag067

**Published:** 2026-06-29

**Authors:** David Leonard, Prashanthi N Thota, Vinay Sehgal

**Affiliations:** Department of Gastroenterology, University College London Hospitals NHS Trust, London, UK; Department of Gastroenterology, Cleveland Clinic, Cleveland, OH, USA; Department of Gastroenterology, University College London Hospitals NHS Trust, London, UK; Department of Gastroenterology, University College London, London, UK

**Keywords:** Barrett’s esophagus, esophageal adenocarcinoma, future perspectives, screening

## Abstract

The incidence of esophageal adenocarcinoma (EAC) has continued to increase, with a poor 5-year survival rate of ~20%. Barrett’s esophagus is the only recognized precursor to EAC, so public health strategies focus on identifying and screening high-risk populations. Currently, the standard screening method is sedated esophagogastroduodenoscopy. However, this approach has challenges, including its invasive nature, need for sedation, higher cost, procedure-related risks, sampling error, and poor suitability for population-wide screening. As a result, alternative screening techniques such as transnasal endoscopy and less invasive options including non-endoscopic cell collection devices with biomarker assays have been explored. In this review, we summarize current approaches for identifying at-risk populations and screening methods and provide insights into technologies that could complement screening strategies in the future.

## INTRODUCTION

The incidence of esophageal adenocarcinoma (EAC) has been rising steadily in Western countries, with more than a six-fold increase in the United States over the past three decades. This trend is linked to rising rates of obesity and gastroesophageal reflux disease (GERD).^1–3^ Despite advancements in therapies, the prognosis for invasive EAC remains poor, with a stagnant 5-year survival rate of ~15–20%.^1^ This poor prognosis is thought in part to be due to a rich submucosal lymphatic network and lack of serosal barrier in the esophagus facilitating early local invasion and metastatic spread. Additionally, many patients present late with symptoms such as dysphagia and weight loss, by which time the disease is often advanced.^1^

**Fig. 1:**
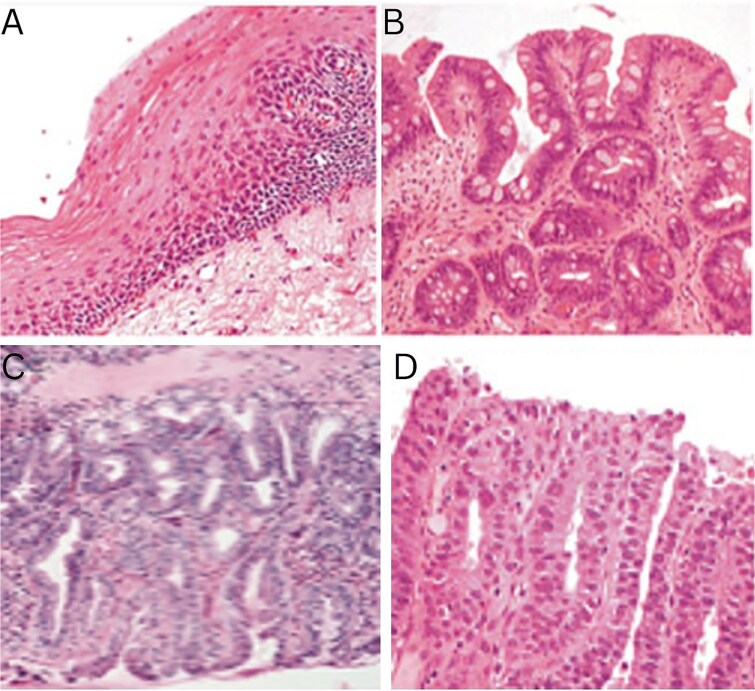
Histological slides showing progression of Barrett’s esophagus.

Barrett’s esophagus (BE), the only known precursor to EAC, it develops when the normal squamous epithelium in the distal esophagus is replaced by columnar epithelium as an adaptive response to chronic GERD.^4^ Endoscopically, BE is characterized by salmon-colored mucosa extending at least 1 cm proximal to the gastroesophageal junction. Most medical societal guidelines agree that the diagnosis of BE requires the presence of intestinal metaplasia (IM), defined as columnar epithelium with goblet cells.^5^ However, this criterion is not required in the current guidelines from the British Society of Gastroenterology (BSG), Asia Pacific region, and Japanese Esophageal Society.^5^^,^^6^ BE is believed to progress to EAC in a stepwise manner, starting from non-dysplastic IM to low-grade dysplasia (LGD), then to high-grade dysplasia (HGD), and finally to EAC (Fig. 1).^2^

Screening an at-risk population for BE allows identified patients to participate in a dedicated surveillance program, though the benefits of this not proven.^7^^,^^8^ Though the potential benefits include the opportunity of offering patients with superficial EAC, HGD, or persistent LGD, endotherapy which potentially could be a curative intervention.

This review aims to provide a comprehensive evaluation of the benefits and limitations of screening for BE. We will first examine the risk factors for BE and outline societal criteria for identifying screening candidates. Subsequently, we will discuss currently available screening modalities and conclude by examining emerging technologies, including blood biomarkers, and breath tests and their prospective integration into future screening paradigms.

## ADVANTAGES AND LIMITATIONS OF SCREENING

Endoscopic screening and surveillance in patients with BE has been associated with detection of earlier-stage cancer and a modest improvement in survival, although this may in part reflect lead-time bias.^8^^,^^9^ An estimated 5% to 6% of adults in the United States have BE, but most cases remain undiagnosed.^10^ This gap highlights limitations in current screening strategies, as sedated esophagogastroduodenoscopy (EGD) is generally reserved for patients with chronic GERD who have additional risk factors.^4^^,^^8^ However, a strategy based on GERD symptoms lacks sensitivity, since up to 40% of patients with EAC report no prior reflux symptoms.^11^ Consequently, fewer than 10% of EAC cases are diagnosed after a prior diagnosis of BE, indicating missed opportunities for early detection.^12^

EAC is an important public health problem and early detection of BE could allow patients to enroll in dedicated surveillance programs, which facilitate the identification of dysplasia or early EAC before it progresses to an advanced stage and thus potentially lead to a reduction in EAC specific mortality.

There is now a large body of evidence to support curative management of superficial neoplasia associated with BE with endoscopic eradication therapy which usually involves resection of visible lesions followed by ablation of the residual BE to prevent recurrence.^13^^,^^14^ Eradication rates of dysplasia with this approach are close to 90% with recent data suggesting this effect is equally durable.^15^ Additionally, with advances and increased uptake of endoscopic submucosal dissection (ESD), BE with suspected superficial submucosal invasion can now also be resected using ESD techniques.^16^^,^^17^

Despite this, population-wide screening for BE is not generally recommended due to its low prevalence in the general population, the substantial costs involved, and the invasiveness of EGD, which remains the gold standard for screening.^18^^,^^19^ As highlighted by the BOSS trial, the benefits of surveillance in BE are unproven.^7^ In this randomized controlled trial of endoscopic surveillance versus at need endoscopy in 3453 patients with BE (maximal histology LGD) with a median follow-up 12.8 years, no difference in overall survival was observed between the groups (hazard ratio 0.95; 95% CI 0.82–1.10; *P* = 0.503) Therefore, targeted case finding of high-risk patients using less-invasive approaches then EGD is gaining increasing attention by most GI/endoscopy societies.^4^^,^^12^^,^^16–18^

## WHO TO SCREEN?

### At-risk population

There are several accepted risk factors for BE which include GERD, smoking, obesity, advancing age (over 50), family history, presence of hiatal hernia, and non-Hispanic White ethnicity.^1^

#### Chronic GERD and hiatal hernia

Chronic GERD is recognized as a key risk factor for the development BE. The majority of professional societies require the presence of chronic GERD as a prerequisite before considering screening for BE.

Diagnosing GERD can be difficult, typical symptoms such as heartburn and regurgitation are often subjective and can also be present in other conditions such as esophageal dysmotility and supragastric belching.^20^^,^^21^ GERD can be diagnosed clinically with a trial of proton-pump inhibitor (PPI), though this approach has a reported sensitivity 78% and specificity 54%, though this approach is limited.^22^ Ambulatory pH monitoring is considered the gold standard, it is highly sensitive (79 to 96%) and specific (85% to 100%) in patients with erosive esophagitis but less so in patients with nonerosive reflux disease.^21^^,^^23^

40%–50% of individuals do not report experiencing chronic GERD prior to their diagnosis and BE is only detected in 10%–15% patients who report having chronic GERD.^18–21^^,^^24–27^ This may be due to patients with BE exhibiting reduced sensitivity to reflux symptoms.^1^

A systematic review and meta-analysis by Eusebi et al. demonstrated a strong association between long-segment BE and chronic reflux, with an odds ratio (OR) of 6.30 (95% CI 2.26–17.61).^24^

The presence of a hiatal hernia has been linked to the development of BE. According to a systematic review and meta-analysis by Ma et al., hiatal hernia is associated with a nearly fourfold increased risk of developing any length of BE (OR = 3.91, 95% CI 3.31–4.62, *P* < 0.001).^28^ The same study showed an even higher risk for developing longer segments of BE in patients with a hiatal hernia (OR = 10.01, 95% CI 4.16–24.06, *P* < 0.001).^28^ As hiatal hernias are very common in patients with severe GERD, the presence of hiatal hernia serves as a surrogate marker for GERD rather than an independent risk factor.

#### Family history of BE/EAC

Having a first-degree relative or family history of BE/EAC is a strong risk factor for BE. In the study by Chak et al., a family history of BE was associated with a 12-fold increased risk of developing BE (OR 12.23, 95% CI 3.34–44.76).^11^ However, it should be noted that this was a small study (*n* = 164), which may limit the generalizability of its findings due to a small sample size. A recent systematic review (*n* = 16,189) reported a modestly increased risk associated with a positive family history: individuals with a family history had a higher likelihood of BE (aRR 3.26; 95% CI 1.43–7.40) and EAC (aRR 2.19; 95% CI 1.14–4.21) compared with those without a family history. There was also evidence of familial clustering in 10% of patients with BE and EAC. Additionally, screening a first-degree relative produced a diagnostic yield between 12% and 44%, which is substantially higher than in the general population.^18^

#### Male sex

Men are more likely than women to develop BE and tend to do so at a younger age.^1^ A systematic review by Eusebi et al. found the pooled prevalence of BE in men with GERD was 10.8% (95% CI 5.6%–15.9%), compared to 4.8% (95% CI 2.7%–7.5%) in women.^24^ EAC is also more common in males than in females with a 8:1 ratio.^29^ These differences may be related to estrogen’s protective effect against GERD-induced esophageal injury.^30^

#### Age

Advancing age is linked to a higher prevalence of BE, Qumseya et al. report a prevalence of BE of 6.1% (95% CI 4.6%–8.1%) of individuals over 50 years in Western populations.^31^ This aligns with the median age of EAC diagnosis in the USA being 68 years, with 79.7% of cases occurring between ages 55 and 84.^32^

#### Ethnicity

Non-Hispanic White ethnicity is linked to a higher risk of developing BE. In a single-center retrospective cross-sectional analysis of 2100 patients undergoing endoscopy, whites had a significantly higher prevalence of BE compared to Hispanics (6.1% vs. 1.7%, *P* = 0.0002) and blacks (6.1% vs. 1.6%, *P* = 0.004).^33^ The reasons for this racial disparity are unclear, and further studies are needed.

#### Tobacco smoking

Tobacco smoking increases the risk of developing BE. In the meta-analysis by Andrici et al., smoking raised BE risk among non-GERD controls (OR 1.44; 95% CI 1.20–1.74) and population controls (OR 1.42; 95% CI 1.15–1.76), but not significantly among GERD controls (OR 1.18; 95% CI 0.75–1.86).^34^ They also observed a dose–response relationship, with higher pack-years of smoking associated with an increased likelihood of developing BE. Krishnamoorthi et al. showed in their meta-analysis that smoking is also linked to a higher risk of progression to HGD/EAC (OR 1.47; 95% CI 1.09–1.98).^35^

#### Central obesity

Central obesity independently increases the risk of developing BE through two main mechanisms: mechanically, by disrupting the GE junction barrier and raising the chances of GERD and hiatal hernia; and metabolically, by promoting pro-inflammatory cytokines which leads to insulin resistance, which in turn increases risks of BE development.^36^ A meta-analysis conducted by Singh et al. found that central adiposity nearly doubles BE risk compared to normal body habitus (adjusted OR 1.98; 95% CI 1.52–2.57).^37^

### Hiatal hernia

#### What do the guidelines say?

There is consensus among various medical societies that targeted screening for BE, with a one-off diagnostic endoscopy, should be offered to patients with established risk factors, however the risk factors to consider differ between societies. Chronic GERD is a pre-requisite for the majority of societies apart from the Committee of the American Gastroenterological Association (AGA) with additional other risk factors. The AGA suggest screening with three established risk factors (Male/Caucasian/age > 50 years/tobacco smoking/chronic GERD/obesity/family history of BE or EAC). The European society of guidelines and American College of Gastroenterology suggest screening in Chronic GERD plus three other established risk factors. The BSG recommends screening in patients with chronic GERD with three established risk factors or family history of BE/EAC. Finally, the American Society Gastrointestinal Endoscopy suggesting screening patients with family history of BE/EAC or history of chronic GERD and one other risk factor. Table 1 summarizes the current recommendations, noting that the BSG and AGA are expected to release updated guidance soon.

**Table 1:** Medical societies recommendations on who to screen

Organization	Recommendation
European Society of Gastrointestinal Endoscopy (ESGE) 2023^16^	**Can be considered in select population:** (***≥50 years of age** with a history of chronic GERD symptoms, **and at least one** of; White ethnicity, male sex, obesity, smoking, having a first-degree relative with BE or EAC*).
Committee of the American Gastroenterological Association (AGA) 2022^82^	**3 established risk factors for BE and EAC:** (*male, non-Hispanic White, Age ≥ 50 years, Smoking History, Chronic GERD, Obesity, Family history of BE or EAC*)
American College of Gastroenterology (ACG) 2022^4^	**Chronic GERD + 3 additional risk factors:** (*male sex, age ≥ 50 years, White race, tobacco smoking, obesity, and family history of BE or EAC in a first-degree relative*)
American Society for Gastrointestinal Endoscopy (ASGE) 2019^83^	If screening endoscopy for BE is performed Screen at risk population: (a) *family history of EAC or BE* or (b) *patients with chronic GERD plus at least 1 other risk factor*
British Society of Gastroenterology (BSG) 2014^46^	**Chronic GERD** symptoms and multiple risk factors: (a) ***3 of the following:** or older, white race, male sex, obesity.* (b) *If family history Barrett’s or EAC*

### Risk stratification

#### Risk prediction models

Several validated risk assessment tools have been developed to help predict the presence of BE by combining multiple risk factors, including chronic GERD. These include the Michigan BE pREdiction Tool (M-BERET),^38^ the Gerson tool,^39^ the Locke tool,^40^ the Thrift tool,^41^ the Nord-Trøndelag Health Study (HUNT),^42^ and the Kunzmann tool^43^ (Table 2). However, it should be noted that only the Thift tool and M-BERET have been independently validated.

**Table 2:** Comparison of risk prediction models for detecting BE

Risk prediction tool	Variables used	AUROC 95% (CI)	Validation
M-BERET	GERD (weekly symptoms), age, waist-to-hip ratio, and smoking (pack-year history)	0.695 (0.625–0.766) 0.72 (0.66–0.78) 0.72 (0.66–0.79)	Comparative study^44^ External validation^84^ Internal validation^38^
Gerson	Symptoms (nocturnal pain, odynophagia, heartburn, dysphagia), male gender, ethnicity	0.692 (0.630–0.753)	Comparative Study^44^
Kunzmann	Age, gender, BMI, smoking, and esophageal conditions (GERD, hiatal hernia, stricture, fundoplication, or on acid suppressive medications)	0.667 (0.595–0.730) ^*^0.80 (0.77–0.82)	Comparative Study^44^ Internal Validation^43^ ^*^5-year risk of developing EAC
HUNT	GERD, age, BMI, smoking, gender	0.665 (0.595–0.730) ^*^0.84 (0.76–0.91)	Comparative Study^44^ ^*^15-year risk of developing EAC^42^
Locke	Chronic GERD (>5 years), age, gender, dysphagia, psychosomatic symptom score, anti-reflux medication	0.660 (0.589–0.731) 0.76	Comparative Study^44^ Internal validation^40^
Thrift	GERD, age, BMI, smoking, gender, anti-reflux medication, and highest level of education	0.61 (0.56–0.66)	External validation^41^

In a prospective study of 1241 patients undergoing their first diagnostic EGD or treatment for early neoplasia in BE, Rubenstein et al. found that each of these tools predicted BE more effectively than using chronic GERD symptoms alone.^44^ Notably, M-BERET, HUNT, and Kunzmann demonstrated the best performance, with area under the curve (AUROC) values of 0.695, 0.680, and 0.708, respectively, compared to an AUROC of 0.579 for chronic GERD symptoms alone (see Table 2). However, these tools are generally not used in clinical practice due to their modest accuracy. In addition, there are no randomized controlled trials that have evaluated these tools. Furthermore, a further limitation is that they rely on information which may not always be available at the time of referral or triage; for example, M-BERET requires waist and hip circumference measurements, as well as a smoking pack-year history.

#### Machine learning models

Machine learning (ML) can help predict BE and EAC using data points widely available from electronic health care records. Rubenstein et al. performed a retrospective case–control analysis of cases diagnosed with EAC or gastric adenocarcinoma (GCA) between 2005 and 2018. They tested the model on 2076 cases, including 2076 cases of EAC with 2,567,069 controls.^20^ Interestingly, when applying the HUNT and Kunzmann prediction tools to the same test subjects, only 840 cases could be analyzed due to missing data points, highlighting a limitation of these models. This further highlight why such models are not widely used in clinical practice.

The researchers validated their tool and found that K-ECAN accurately identified individuals who would develop EAC and GCA up to three years in advance. The model demonstrated better discrimination than other validated models, with an AUROC of 0.77.^20^ Conversely, since the tool was validated only in veterans hospitals, further research is needed to confirm its effectiveness across different demographic groups to ensure generalizability.

Iyer et al. have developed and internally validated two ML models, which were tested on a large database of Mayo Clinic USA patients, including 6 million individuals.^45^ A total of 84,676 BE cases, 1539 EAC cases, and 252,276 control cases were identified. The models incorporated traditional risk factors and also identified additional predictors such as coronary artery disease, serum triglycerides, and electrolyte levels. The BE model demonstrated both sensitivity and specificity of 76%, with an AUROC of 0.84.^45^ The EAC model showed a sensitivity of 84%, specificity of 70%, and an AUROC of 0.84.^45^ These results outperformed existing risk prediction scores. However, before these models can be widely implemented in clinical practice, further validation using real-time data across different electronic health record platforms is necessary to ensure their reliability and adaptability.

## CONVENTIONAL EGD

Sedated EGD is regarded as the gold standard technique for BE screening. This involves inspecting the esophagus with high-definition white light (HD-WLI), assessing the length of the BE segment, and noting the presence of a hiatus hernia in accordance with the Prague classification.^1^^,^^46^ The Seattle protocol is a standardized biopsy approach whereby quadrantic biopsies are taken every 2 cm along the Barrett’s segment, with additional targeted biopsies performed on visible lesions.^46^ The sensitivity and specificity of EGD have been reported at 92% and 100%, respectively.^47^

Additionally, the use of virtual chromoendoscopy, can enhance mucosal visualization and improve detection of dysplasia and potentially reduce the number of biopsies needed.^47^ However, EGD does have limitations, including inter-operator variability in quality, the invasiveness of the procedure, frequent need for sedation due to potential discomfort, and high procedural costs.^1^

Despite these limitations, several cost-effectiveness analyses, such as those by Inadomi et al., have demonstrated that screening EGD in select populations is more cost-effective than no screening.^48^^,^^49^ Detecting neoplasia in BE remains a significant clinical challenge, particularly as subtle or high-risk lesions are frequently missed during index EGD. A systematic review and meta-analysis of 820 EAC patients, revealed that 25% of EAC were ‘missed,’ that is, these patients had an EGD within one year showing only non-dysplastic BE prior to their EAC diagnosis.^50^ These findings highlight the importance of ensuring quality and the need for high-quality performance indicators for BE screening and surveillance.^51^

Artificial intelligence and computer-aided detection (CADe) tools could assist and help with quality and detection of Barrett’s-associated neoplasia.^52^ Notably, the study by Fockens et al. introduced a CADe system that substantially increased the sensitivity for neoplasia detection among general endoscopists from 74% to 88% (OR 2.04; 95% CI 1.73–2.42; *P* < 0.0001), achieving performance comparable to expert endoscopists.^53^ Despite these advances, a key limitation is that many AI systems are developed and validated under ideal conditions using images and videos sourced from expert centers, which may restrict their generalizability to broader clinical practice. As far as we are aware, no AI-assisted endoscopic systems to improve BE detection in patients without known BE are yet published.

## NON-ENDOSCOPIC CELL COLLECTION DEVICES

**Fig. 2:**
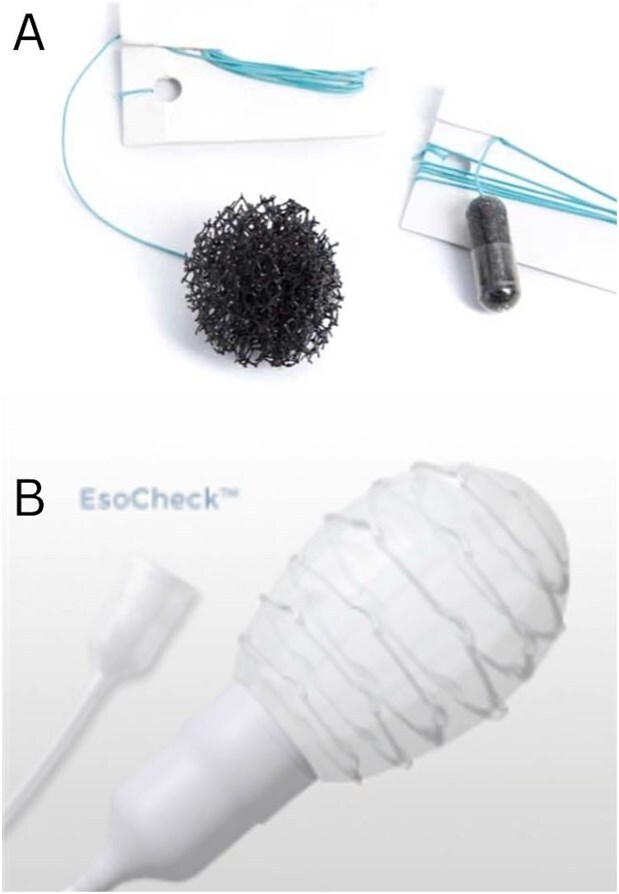
Images of non-endoscopic cell collection devices (CCDs).

Details and relevant studies are in Table 3, with images in Fig. 2.

**Table 3:** Comparison of non-endoscopic cell collection devices (CCDs)

Cell collection device (year)	Biomarker	Study	Sensitivity	Specificity	Adverse events
Cytosponge 30 mm sponge (2010)^85^	TFF3	Prospective cohort study (*n* = 504)	73.3% (short segment 1–2 cm) 90.0% (long segment ≥2 cm)	93.8% (short segment 1–2 cm) 93.5% (long segment ≥2 cm)	3 patients unable to swallow capsule 2 sponges failed to expand and low cell yield
Cytosponge 30 mm sponge (2015)^55^	TFF3	Case–control study(*n* = 1110) *463 controls* *647 BE cases*	79.9%	92.4%	16.7% of patients had oozing of blood from a Cytosponge abrasion site no intervention needed
Cytosponge 30 mm Sponge (2020)^56^	TFF3	RCT (BEST-3) (*n* = 13,222) 6834 intervention group ➔ 1654 swallowed capsule 6388 usual care	-	-	96 unable to swallow Cytosponge 1 case of sponge detachment 1 sore throat 2 Esophageal and gastric pain
EsophaCap 25 mm Sponge(2018)^86^	2 MDM	Pilot Study (*n* = 41) *21 controls* *20 BE cases*	100%	100%	2% unable to swallow device
EsophaCap 25 mm Sponge (2020)^87^	5 MDM	Multisite case control study (*n* = 201) *89 controls* *112 BE cases*	92%	94%	Nil
EsophaCap 25 mm Sponge (2021)^58^	5 MDM	Multi-center case control study: Training set using 5 MDM (*n* = 199) *89 controls* *110 BE cases* Test set using 5 MDM (*n* = 89) *29 controls* 60 BE cases ^*^Also tested on multiple 3 MDM combinations achieving similar results to 5 MDM	93% (Training set) 93% (Test set)	90% (Training set) 93% (Test set)	1 case of sponge detachment—sponge removed endoscopically
EsophaCap 25 mm Sponge (2024)^57^	3 MDM	Multi-center cohort study: Training set using 5 MDM (*n* = 199) *89 controls* *110 BE cases* Test set using 5 MDM (*n* = 89) *29 controls* *60 BE cases*	82% (Training set) 88% (Test set)	90% (Training set) 84% (Test set)	1 case of sponge detachment 2 sponges failed to expand
EsoCheck 16 × 9 mm capsulated balloon (2018)^88^	2-marker panel: mVIM and mCCNA1 DNAs	Pilot Study *N* = 86	90.3%	91.7%	18% unable to swallow device
EsoCheck Swallowable catheter with encapsulated balloon	EsoGuard assay (mCCNA1, mVIM)	Multicenter prospective trial (*n* = 243) *155 controls* *88 BE cases*	85%	85% 84% (NDBE)	None reported

### Cytosponge

Cytosponge has been the most researched CCDs to date and relies on a sponge-based approach. The device consists of a capsule tethered to a thread, which is swallowed and dissolves (over 5–7 minutes) in the stomach releasing a 30 mm polyurethane sponge.^54^ This sponge is then withdrawn, collecting esophageal samples containing over a million cells. The collected cells are analyzed using immunohistochemical staining for trefoil factor 3 (TFF3) expression, a biomarker for IM.

Several trials have assessed the efficacy and safety of Cytosponge. The BEST2 case–control study involved 1100 participants (463 GERD controls and 647 confirmed BE cases) from 11 UK hospitals, where patients underwent Cytosponge prior to endoscopy. The test achieved a sensitivity of BE detection of 79.9% and specificity of 92.4%, this increased to 87% in cases with BE length > 3 cm.^55^

The BEST3 trial was a randomized controlled study conducted at 109 general practice clinics in the UK. Patients over 50 with at least 6 months of acid-suppressant use were eligible and randomized to receive either standard care or offered a Cytosponge-TFF3 test (initially randomization was cluster based by clinic, but lately changed to individual based).^56^ Cytosponge led to a tenfold increase in BE diagnoses, despite a modest uptake of 24% compared to standard care (acid suppression and at-need endoscopy). The Cytosponge group also detected more cases of BE-associated dysplasia, but the numbers for this were small. An economic evaluation using data from the BEST-3 trial found that Cytosponge was likely cost-effective in comparison to usual care.^56^ They showed that a one-off screen with Cytosponge in patients with a median age of 69 years cost an additional £82 per patient and generated 0.015 QALYs compared to usual care, resulting in an ICER of £5500 per QALY gained.^56^

### EsophaCap

The EsophaCap like the Cytosponge device consists of a swallowable capsule sponge, which is attached to a string. The sponge is smaller than the Cytosponge at 25 mm.^57^ The samples collected are combined with methylated DNA markers, which are markers associated with BE metaplasia and dysplasia.

The benefit of this technique, in comparison to using TFF3, is that quantitative PCR can be performed in an automated fashion, whereas TFF3 requires a pathologist to perform immunohistochemistry. This makes the method more scalable. The device has been used with panels of 2, 3, and 5 biomarkers.^58^

More recently, a novel three-biomarker panel was discovered, identifying three key markers: USP44, TBC1D30, and NELL1.^59^ It was tested on a cohort of 199 patients and independently on 35 patients. The panel was able to accurately differentiate between HGD or EAC and non-BE controls with an AUROC of 0.969 (95% CI 0.911–1.00) and was able to distinguish NDBE from controls with an AUROC of 0.82 (95% CI 0.748–0.889).^59^

### EsoCheck

The EsoCheck is a swallowable encapsulated balloon that is ingested and then inflated in the stomach with air. Once inflated, the balloon is slowly withdrawn up to 5 cm so that its textured surface collects cells from the distal esophagus. After collecting cells from the esophagus, the balloon is deflated and inverted into the capsule for safe removal. The entire procedure typically takes around 2–3 minutes.^60^

The collected cells are then subjected to DNA extraction and analyzed using the EsoGuard assay, which detects the aberrant methylation of cyclinA1 (CCNA1) and vimentin (VIM) genes indicative of BE. In an independent prospective multicenter trial, EsoCheck demonstrated a sensitivity of 85% and a specificity of 90% for detecting BE.^60^ However, some challenges have been reported with the device, including inability to swallow by 17% of subjects and insufficient DNA sample extraction in 14% of cases.^60^

A larger retrospective multicenter study involving 11,991 participants showed successful cell collection in 94.7% of cases, with an average procedure time of under 2 minutes and no adverse events.^61^ These findings indicate excellent tolerability and safety of the device.

## OTHER IMAGING TECHNIQUES

### Transnasal endoscopy

Unsedated transnasal endoscopy (TNE) is an endoscopic method that involves passage of an ultra-thin endoscope through the nares. This less invasive technique can be performed outside the endoscopic department, such as in an outpatient office or a mobile van.^1^^,^^17^ The sensitivity for detecting BE is generally comparable to that of EGD, with sensitivities above 90%. In a systematic review and meta-analysis by Huibertse et al., TNE demonstrated a pooled sensitivity between 98% and 99%, with comparable tolerability to EGD and a technical success rate ranging from 89% to 100%.^62^ These results indicate that TNE is both highly sensitive for detecting BE and well-tolerated by patients, making it a strong alternative to traditional EGD for screening purposes. Overall, patient tolerability of TNE was higher or equal compared with EGD, and the technical success rate of TNE ranged from 89% to 100%.^62^ Sami et al., in their randomized controlled trial, reported that over 80% of patients were willing to have further TNE in the future, showing generally high acceptability among patients, which is useful when encouraging uptake for screening.^63^ Various cost-effectiveness studies have shown that TNE is more cost-effective than sedated EGD. For example, Nietert et al. demonstrated that TNE, compared to no screening, had an incremental cost-effectiveness ratio (ICER) of $55,740 per quality-adjusted life year (QALY) gained, whereas EGD had an ICER of $86,833.^64^ However, due to the small working channel, typically around 2 mm in diameter, only small biopsies can be taken.^65^ Additionally, the optical resolution of a TNE scope is not as good as EGD, so it is not recommended for in-depth optical diagnosis or surveillance.^17^

### Tethered capsule endomicroscopy

Tethered capsule endomicroscopy (TCE) is an advanced imaging technique that utilizes optical coherence tomography (OCT) to deliver high-resolution, cross-sectional images, enabling microscopic visualization of the entire esophagus.^66^ This is particularly useful, as highlighted by a meta-analysis of patients who underwent esophagectomies for high-grade dysplasia, which found that 13% actually had invasive cancer that was not identified during initial biopsies; thus, despite protocolled biopsies, advanced disease is not always detected.^67^ Unlike traditional OCT technologies that are restricted to small segments, TCE allows for a comprehensive assessment of the whole esophagus. The procedure involves swallowing a tethered capsule and can be conducted in an outpatient setting, similar to TNE and other swallowable capsule devices. A multicenter feasibility study involving 147 patients demonstrated that TCE consistently measures BE segment length and detects dysplasia, with findings correlating well with subsequent EGD results. High-quality images were obtained in 93.7% of cases, and patients reported good tolerability.^68^ The combination of reliable imaging and patient comfort supports the wider adoption of TCE in clinical practice. However, expert training is currently required to interpret the images, and a notable limitation is the inability to obtain confirmatory tissue samples for diagnosis.

### Esophageal capsule endoscopy

Esophageal capsule endoscopy (ECE) is similar to the already widely used small bowel and colonic capsule endoscopy, but utilizes a dedicated capsule designed specifically for the foregut and esophagus such as the PillCam ESO; Medtronic.^69^ This wireless capsule contains a camera, battery, and radio transmitter, which transmits images to a digital receiver. The procedure is well tolerated by patients and can be performed in an outpatient setting.^70^ It also has the opportunity to survey other parts of the GI tract on the same sitting.^19^

One drawback is that while BE can be identified, additional procedures would be necessary to collect samples, which would reduce the cost-effectiveness. A cost-effectiveness analysis by Reubenstein et al. reported that sEGD cost $1998 per patient and provided 16.66 QALYs, compared to $2392 and 16.64 QALYs for ECE.^71^ Additionally, ECE has lower sensitivity compared to EGD. Bhardwaj et al., in their meta-analysis, found that ECE had a pooled sensitivity of 78% and a specificity of 86%.^70^ The lower sensitivity of ECE is mainly due to the rapid esophageal transit, which can hinder optimal image capture. This issue may be addressed by a newer approach, magnetically assisted capsule endoscopy (MACE).^72^ With MACE, a swallowable capsule is maneuvered within the esophagus using external magnets. A pilot study by Beg et al. showed that MACE is safe and effective, achieving high accuracy for detecting BE, though further research is needed to validate these results.^72^

## OTHER EMERGING NON-INVASIVE TECHNIQUES

### Exhaled volatile organic compounds

Breath analysis, via mass spectrometric analysis, has emerged as a promising, non-invasive approach for cancer detection, including BE/EAC and other malignancies.^66^ This technique focuses on identifying volatile organic compounds (VOCs), which are metabolic byproducts produced by both human cells and the gut microbiome.^73^ Disease states can alter the profile of these exhaled compounds, making volatile organic compound analysis a potentially valuable diagnostic tool. The drawback of mass spectrometry is that it is costly and labor intensive.

An electronic nose device offers an alternate analytical method, which is portable, user-friendly, and potentially cost-effective solution that could facilitate large-scale population screening. This technology uses a series of metal oxide sensors to measure electrical conductivity profiles in response to interactions with VOCs. A 32-point thermal cycle is applied to maximize these interactions; the resulting output is then analyzed to create a ‘breath print,’ which can potentially be used to estimate disease states such as BE.^74^^,^^75^ In a proof-of-concept study by Peters et al., breath analysis using the electronic nose device Aeonose, was able to diagnose BE with a sensitivity of 91% (95% CI 84%–95%), specificity of 74% (95% CI 69%–79%), and an AUROC of 0.91 (95% CI 0.87–0.94).^76^ Despite these encouraging results, further research is necessary to determine how factors such as GERD, PPI use, and other medical conditions may influence breath analysis outcomes.

### Microbiota

There is growing evidence that alterations in oral and esophageal microbiome may be involved in the development of BE and EAC.^77^^,^^78^ In a healthy esophagus, there is generally a diverse microbiota, mainly derived from oral flora with a predominance of gram-positive bacteria, especially species from the streptococcal genus. In contrast, patients with BE tend to have a less diverse microbiota, with a higher proportion of disease-associated gram-negative bacteria, which are often linked to inflammation and may contribute to disease progression.^4^ Sampling the oral microbiome is a non-invasive, feasible, and reproducible process that can be performed using various methods, such as saliva collection or oral mouth rinses.^79^ Cytosponge has also been investigated as a reliable method for collecting esophageal samples.^80^

Snider et al. conducted a case–control study involving 49 patients (32 with BE and 17 controls) scheduled for endoscopy.^78^ Saliva, oral, and esophageal samples were collected. At the phylum level, the oral microbiome demonstrated an increase in Firmicutes (*P* = 0.005) and a decrease in Proteobacteria (*P* = 0.02). They also observed a higher abundance of Enterobacteriaceae in HGD and EAC patients compared to NDBE, which could have a potential value in surveillance. In a nested control study (81 with EAC and 160 controls), patients swished 10 mL of mouthwash and the sample was assessed using 16S rRNA gene sequencing.^77^ They identified *Tannerella forsythia*, a periodontal pathogen linked to a higher risk of developing EAC.^77^

This is an exciting field and more data is needed to understand how factors such as diet, medications such as PPIS can influence microbiome diversity. Future research could focus on therapeutic manipulation of the microbiota.

### Liquid biopsy

MicroRNAs (miRNAs) are small non-coding RNA molecules that represent promising non-invasive biomarkers, which can be sampled from peripheral blood. MiRNAs play a key role in regulating gene expression, as well as cell growth and differentiation.^19^ Several specific miRNAs become dysregulated in patients with BE and EAC. The EMERALD study, a retrospective international multicenter study, reported promising results for a new 6-miRNA panel with high diagnostic accuracy for early detection of BE and EAC (AUROC: 91.9%, sensitivity: 82.5%, specificity: 90.5%).^81^ The biomarkers in this panel include miR-15a, miR-18a, miR-21, miR-106b, miR-146a, and miR-93, the latter three of which have been identified as non-invasive biomarkers in previous studies.^81^ The test demonstrated a specificity of 95.2% for a normal esophagus, a sensitivity of 80.6% for detecting BE with or without LGD, and a sensitivity of 95.8% for detecting EAC or BE with HGD.^81^

## CONCLUSION

EAC remains a highly lethal malignancy, largely due to late-stage diagnosis in most patients. BE is widely accepted as its principal precursor lesion, yet the majority of cases remain undetected. Current screening strategies rely on symptom-based selection and sedated endoscopy, which limits population-level uptake. Consequently, a substantial proportion of at-risk individuals are never evaluated. Emerging data support a shift toward risk-stratified screening models that extend beyond GERD-based criteria. Electronic medical records integrated with AI-based tools may enable automated identification of individuals at increased risk for BE, improving case-finding and targeting of screening. Minimally invasive, office-based sampling tools, such as Cytosponge and EsoCheck, offer scalable alternatives to endoscopy. These technologies, combined with molecular biomarkers, improve the ability to detect BE and identify higher-risk disease. Advances in DNA methylation and other molecular signatures further enhance non-endoscopic screening approaches. Future strategies are likely to integrate AI-enabled risk prediction within electronic health records with minimally invasive testing and potentially mass screening models that do not require physician-led procedures, forming a two-step diagnostic pathway. This evolving framework has the potential to improve early detection and ultimately reduce mortality from EAC.

## Notes

Specific author contributions: David Leonard (Writing—original draft, Writing—review & editing), Prashanthi Thota (Methodology, Supervision, Writing—original draft, Writing—review & editing), Vinay Seghal (Methodology, Supervision, Writing—original draft, Writing—review & editing).

Financial disclosure: The authors have no financial disclosures to make. There were no conflicts of interest.

Financial support: None.

Potential competing interests: None.
